# Nuciferine Inhibited the Differentiation and Lipid Accumulation of 3T3-L1 Preadipocytes by Regulating the Expression of Lipogenic Genes and Adipokines

**DOI:** 10.3389/fphar.2021.632236

**Published:** 2021-03-22

**Authors:** Hanyuan Xu, Linjie Wang, Kemin Yan, Huijuan Zhu, Hui Pan, Hongbo Yang, Meijuan Liu, Fengying Gong

**Affiliations:** Key Laboratory of Endocrinology of National Health Commission, Department of Endocrinology, Peking Union Medical College Hospital, Chinese Academy of Medical Sciences and Peking Union Medical College, Beijing, China

**Keywords:** nuciferine, differentiation, proliferation, adipokines, fatty acid synthase, 3T3-L1 preadipocytes

## Abstract

**Purposes:** Nuciferine, a main aporphine alkaloid component found in lotus leaf (*Nelumbo nucifera*), has been demonstrated to possess the property of reducing fat mass and alleviating dyslipidemia *in vivo*. The purpose of this study is to explore the effects of nuciferine on the proliferation and differentiation of 3T3-L1 cells and further investigate the possible underlying molecular mechanisms.

**Methods:** 3T3-L1 preadipocytes were treated with 0∼20 μM nuciferine for 24∼120 h, the cell viability was assessed using CCK8. 3T3-L1 preadipocytes and human primary preadipocytes were then induced differentiation and the effects of nuciferine on the lipid metabolism in differentiating and fully differentiated adipocytes were observed by the methods of intracellular triglyceride (TG) assay, Oil Red O staining, RT-qPCR and western blot. Transient transfection and dual luciferase reporter gene methods were used to assess the effects of nuciferine on FAS promoter activities.

**Results:** Nuciferine inhibited the proliferation of 3T3-L1 preadipocytes in a dose- and time-dependent manner. 20 μM nuciferine significantly attenuated lipid accumulation and reduced intracellular TG contents by 47.2, 59.9 and 55.4% on the third, sixth and ninth day of preadipocytes differentiation, respectively (all *p* < 0.05). Moreover, the mRNA levels of PPARγ, C/EBPα, C/EBPβ, FAS, ACC, HSL and ATGL were notably decreased by 39.2∼92.5% in differentiating preadipocytes when treated with 5∼20 μM nuciferine (all *p* < 0.05). In fully differentiated adipocytes treated with 20 μM nuciferine for 48 h, the mRNA levels of FAS, ACC and SREBP1 were remarkably downregulated by 22.6∼45.2% compared with the controls (0 μM) (all *p* < 0.05), whereas the expression of adipokines FGF21 and ZAG were notably promoted by nuciferine. Similarly, in fully differentiated human primary adipocytes, the mRNA levels of FAS, ACC, SREBP1 were decreased and the expression of FGF21 and ZAG were elevated after treated with nuciferine (all *p* < 0.05). Further mechanism studies showed that 2.5∼20 μM nuciferine significantly decreased FAS promoter activities in 3T3-L1 preadipocytes.

**Conclusion:** Nuciferine inhibited the proliferation and differentiation of 3T3-L1 preadipocytes. The inhibitory effects of nuciferine on adipogenesis might be due to the downregulation of PPARγ, C/EBPα and C/EBPβ, which led to the reduction of intracellular lipid accumulation in 3T3-L1 cells and by downregulating the expression of critical lipogenic enzymes, especially of FAS, which was achieved by inhibiting the FAS promoter activities. Besides, nuciferine promoted the expression of adipokines in fully differentiated adipocytes.

## Introduction

Obesity is a wide spread chronic disease which is becoming one of the major risk factors for various diseases including cardiovascular diseases, type 2 diabetes and tumors. It is characterized by excessive triglyceride (TG) accumulation resulting from increased food intake and/or lessened energy consumption. In obesity states, as excessive energy is stored in the form of fat within the body, the volume of adipose tissue mass is enlarged (Longo et al., 2019). It’s well-known that preadipocytes are capable of propagating and differentiating into mature adipocytes and the intracellular TG contents determines the size of adipocytes. Therefore, the number and the size of mature adipocytes reflects the overall volume of fat mass (White and Ravussin, 2019). Fat depots expands by increasing the number of adipocytes and the size of average fat cell volume, thus regulating proliferation and differentiation of preadipocytes establishes a new therapeutic target for obesity and related complications (White and Ravussin, 2019). In the present study, 3T3-L1 preadipocyte line was selected as it is known to be one of the most characterized cell lines used in studying the molecular mechanism involved in the proliferation and differentiation of adipocytes (Liu et al., 2017).

Fat flows through dynamic changes in the synthesis and breakdown of triglycerides in adipocytes. During which a number of key transcription factors are involved. Peroxisome proliferator-activated receptor γ (PPARγ), CCAAT/enhancer binding protein *α* and β (C/EBPα, C/EBPβ) are cardinal transcription factors regulate the expression of adipogenesis-related genes in differentiating preadipocytes. C/EBP β was induced initially upon differentiation stimuli, then C/EBPα and PPARγ were induced to cooperatively promote adipogenesis alongside the process of differentiation. It is asserted that the activation of these factors brings about mitotic arrest, whereafter initiates the differentiation of preadipocytes and maintains the process (Feng et al., 2016). At late phase of differentiation, along with the increased expression of sterol-regulatory element binding protein 1 (SREBP1), a key transcription factor of fatty acid synthesis, the expression of downstream enzymes including fatty acid synthase (FAS) and acetyl-CoA carboxylase (ACC) are activated to promote adipogenesis (Auwerx et al., 1996; Kim and Spiegelman, 1996). Together, the expression of hormone sensitive lipase (HSL) and adipose triglyceride lipase (ATGL) are gradually increasing to participate in the dynamic of lipid biosynthesis and breakdown. Studies have shown that altered expression of these key factors and enzymes heavily contributes to the aggravation of fat amassing and lead to adipose tissue dysfunction (Langin, 2006; Lenhard, 2011; Bolsoni-Lopes and Alonso-Vale, 2015).

Increasingly abundant evidence has demonstrated that adipose tissue serves not only as an energy storage organ, but also as a crucial endocrine organ in our body (Kershaw and Flier, 2004). It is found that adipose tissue has the ability of secreting over 600 different kinds adipokines, which are closely involved in helping coordinate lipid metabolism and contribute to maintaining metabolic homeostasis (Kahn et al., 2019). Zinc-α2-glycoprotein (ZAG) and fibroblast growth factor 21 (FGF21) were reported to be two crucial adipokines which are responsible for regulation of a variety of biological processes including cell proliferation, differentiation and cellular lipid accumulation (Babaknejad et al., 2018; Severo et al., 2020). Among which ZAG is a 42-kDa secretory protein that promotes lipid degradation and regulates insulin sensitivity. In particular, our previous studies showed that ZAG was involved in regulation of body weight through inhibition of lipogenic enzymes in adipose tissue and liver (Gong et al., 2010; Liu et al., 2018), and ZAG treatment suppressed the differentiation and lipid accumulation in 3T3-L1 preadipocytes (Gong et al., 2009). FGF21, mainly produced by liver and adipose tissue, was reported to stimulate the oxidation of fatty acids and inhibit lipogenesis. Besides, it also induced thermogenic effect in brown adipose tissue and led to significant weight loss (Sonoda et al., 2017; Severo et al., 2020). Thus, given their close association with lipid metabolism, the two adipokines may serve as potential therapeutic targets for pharmacological intervention in obesity.

*Nelumbo nucifera* leaf, commonly known as lotus leaf, embraces a long history being used as a medicine for hyperpyrexia, hyperglycemia and hyperlipidemia for hundreds of years in southeastern Asia. The efficacy and safety of lotus leaf extracts in treating obesity and related metabolic disorders have also been reported (Du et al., 2010). Previous experiments conducted in our laboratory showed that lotus leaf extracts significantly reduced visceral fat mass and improved insulin sensitivity in HFD-induced obese rats (Yan et al., 2017). Recent studies further revealed that the pharmacological actions of lotus leaves are mainly attributed to nuciferine, a major active aporphine that was recorded as one of the main active components of lotus leaves (Sharma et al., 2017). It is reported that nuciferine possesses the properties of regulating glucose and lipid metabolism including promoting insulin secretion (Nguyen et al., 2012), inhibiting lipogenesis and attenuating inflammations (Zhang et al., 2015). Results of recent research found that nuciferine is capable of alleviating dyslipidemia and hepatic steatosis in high-fat diet (HFD)-fed hamsters (Guo et al., 2013), and similar results were obtain in streptozocin-induced diabetic mice, as nuciferine treated mice exhibited decreased hepatic levels of total cholesterol, TGs and the number of intracellular lipid droplets (Zhang et al., 2018). Moreover, *in vitro*, nuciferine attenuated fatty acid-induced hepatic steatosis in HepG2 hepatocytes (Zhang et al., 2015; Zhang et al., 2018). Additionally, Ma, et al. found that nuciferine decreased the number of lipid droplets and TG contents in mature adipocytes (Ma et al., 2015), and in differentiating preadipocytes cultured in presence with nuciferine, adipogenic factors were found to be decreased after 8 days of differentiation (Yoo et al., 2019). However, the effect of nuciferine in preadipocytes on early, middle and late phases of differentiation remains unclear and there’re only limited studies thoroughly investigating how different dosages or action times of nuciferine act on differentiating preadipocytes and fully differentiated adipocytes and its related mechanism.

In all, nuciferine possesses the ability of inducing weight loss and bring about metabolic benefits. During the development of obesity, both active cell growth and morbidly enlarged adipocytes contributes to the expansion and dysfunction of adipose tissue, but whether nuciferine ameliorate obesity through regulating proliferation and differentiation of preadipocytes remains unclear. As murine and human *in vitro* models have been extensively utilized in studying lipid metabolism, we used both murine 3T3-L1 and human primary preadipocytes to explore the effects of nuciferine on the proliferation and differentiation of preadipocytes and to investigate its possible mechanisms.

## Material and Methods

### Cell Culture and Cell Experiments of 3T3-L1 and Human Primary Preadipocytes

**FIGURE 1:**
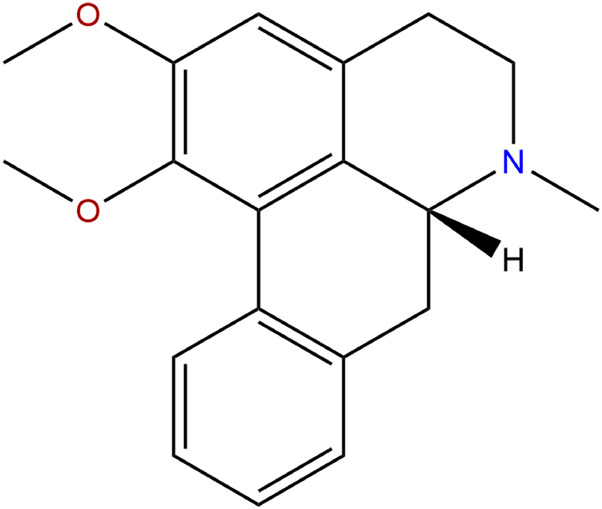
Molecular structure of nuciferine.

The murine 3T3-L1 preadipocytes were cultured according to protocol previously implemented in our lab (Zhu et al., 2013; Zhu et al., 2015; Yan et al., 2020). Briefly, 3T3-L1 cells were cultured in DMEM/F12 medium supplemented with 10% FBS and 1% antibiotics (100 U/ml penicillin and 100 U/ml streptomycin) in CO_2_ incubator (Thermo, United States). Human primary preadipocytes were obtained from the visceral adipose tissue of an obese patient (female, 21-year-old, BMI = 35.5 kg/m^2^) undergone laparoscopic sleeve gastrectomy. The study was approved by the ethics committee of Peking Union Medical College Hospital (No. JS-1093). Patient provided informed consent before adipose tissue was obtained during the surgery. Human visceral adipose tissue (about 8 g) was obtained, rinsed with 1x phosphate buffered saline (PBS) for three times and minced with scissors. 8ml of type I collagenase (2 mg/ml) (Life Technologies, Van Allen Way, CA, United States) were used to digest the adipose tissue, and equal amount of basal culture medium (DMEM/F12 medium supplemented with 10% FBS, 1% antibiotics, 33 μM biotin, 17 μM pantothenic acid and 10 μg/ml transferrin, Sigma, St. Louis, MO, United States) were added to terminate the digestion. Samples were then filtered through a 100 μm mesh and centrifuged at 600 g for 5 min. The supernatant was disposed and the cells at the bottom were resuspended in basal culture medium, then cultured in CO_2_ incubator. The growth state of cells was observed under an inverted phase-contrast microscope (Nikon eclipse Ti, Nikon Corporation, Japan). Nuciferine (purification ≥98% by HPLC) was obtained from Biopurify Phytochemicals Ltd (Chengdu, China) and diluted in dimethyl sulfoxide (DMSO, SolarBio, China) for cell experiments. Its molecular weight was 295.38 and the formula was C19H21NO2. The molecular structure was displayed in Figure 1.

### The Effects of Nuciferine on the Proliferation of 3T3-L1 Preadipocytes

3T3-L1 preadipocytes were plated in 96-well plate with a density of 1 × 10^3^/well. After 24 h, the medium was changed to 10% FBS DMEM/F12 with nuciferine at different concentrations (0, 2.5, 5, 10 and 20 μM). The plates were then rinsed with 1 × phosphate buffered saline (PBS) twice after cells were treated with nuciferine at different concentrations (0, 2.5, 5, 10 and 20 μM) for different action times (24, 48, 72, 96 and 120 h). Cells in control group were treated with equal amount of vehicle (DMSO) for the same time. Cell growth rate was determined by Cell Count Kit-8 (CCK-8, MedChem Express, HY-K0301, United States) by adding 10 μL 2-(methoxy-4-nitrophenyl)-3-(4-nitrophenyl)- 5-(2,4-disulfophenyl)2H-tetrazolium (WST-8) solution into each well and cultured for 4 h in the incubator. Finally, optical density (OD) value was obtained by using a spectrophotometer (Thermo, United States) at a wavelength of 450 nm.

### The Effects of Nuciferine on the Differentiation of 3T3-L1 Preadipocytes and Human Primary Preadipocytes

Differentiation of 3T3-L1 preadipocytes and human primary preadipocytes were conducted in our lab as described previously (Zhu et al., 2013; Zhu et al., 2015; Miao et al., 2019; Yan et al., 2020). Briefly, 2 days after preadipocytes achieved confluence (designated as day 0), differentiation was induced by culturing cells with 10% FBS DMEM/F12 medium (for 3T3-L1 preadipocytes) or basal culture medium (for human primary preadipocytes) supplemented 1 mg/ml insulin, 50 mM 3-isobutyl-1-methylxanthine (IBMX) and 10 mM dexamethasone (Gibco BRL, Grand Island, United States) for 4 days. The differentiation medium was replaced with 10% FBS DMEM/F12 medium or basal culture medium supplemented 1 mg/ml insulin (day 5) for another 2 days. The medium was then changed to 10% FBS DMEM/F12 or basal culture medium (day 7) afterward and kept refreshing every 2 days.

#### Oil Red O Staining and Triglyceride Contents Determination

3T3-L1 preadipocytes were plated at a density of 2 × 10^4^/well in 24-well plates and the differentiation was induced as described above. Cells were then treated with 0, 5, 10 and 20 μM nuciferine for 3, 6, 9 days, respectively. Oil Red O staining was conducted afterward. Firstly, the cells were fixed with 10% fresh formaldehyde for 1 h at room temperature, followed by staining with 0.6% (w/v) filtered Oil red O solution (Thermo, United States) for 2 h. Stained lipid droplets were visualized and photographed by an inverted contrast microscope (Nikon eclipse Ti, Nikon Corporation, Japan). Then 600 μL isopropanol was added to extract oil red O dyes, and 150 μL extracted solution was used for OD value detection using a spectrophotometer (Thermo, United States) at a wavelength of 492 nm. As for the determination of cellular TG contents, the cells were treated with 0, 5, 10 and 20 μM nuciferine for 3, 6 and 9 days, respectively, then lysed and the OD value were measured followed the instructions of commercial triglycerides GPO-POD enzymatic assay kit (Comin Biotechnology, Suzhou, China). The cell total protein concentration was measured using a BCA Protein Assay Reagent kit (Beyotime Biotechnology, China). Intracellular lipid contents were normalized against total protein contents.

#### Real-Time Fluorescence Quantitative PCR Analysis

3T3-L1 preadipocytes and human primary preadipocytes were plated at a density of 2 × 10^4^/well in 24-well plate, then were induced to differentiation as described in 2.3. For the detection of PPARγ, C/EBPα, C/EBPβ, FAS, ACC, HSL, ATGL mRNA levels in the differentiating 3T3-L1 preadipocytes, the cells were treated with nuciferine (0, 5, 10 and 20 μM) for 3, 6 and 9 days, respectively. For the detection of FAS, ACC, SREBP1, HSL, FGF21 and ZAG mRNA levels in fully differentiated murine adipocytes (day 9), the cells were treated with nuciferine (0, 2.5, 5, 10 and 20 μM) for (0, 24, 48, 72 and 96 h). For the detection of FAS, ACC, SREBP1, HSL, FGF21 and ZAG mRNA levels in fully differentiated human primary adipocytes, the cells were treated with nuciferine (0, 2.5, 5, 10 and 20 μM) for 48 h. RT-qPCR was performed with SYBR green fluorescent dye using an ABI7500 PCR system (Applied Biosystems, Beverly, MA, United States) as previously described (Yan et al., 2020; Zhu et al., 2016). In brief, the total RNA of cells was isolated by using E. Z.N.A Total RNA Kit I (Omega Biotek United States). 1μg of total RNA was used for inverse transcription by PrimeScript™ RT reagent Kit (Takara Bio, Kyoto, Japan) and Oligo (dT) primer. The primer sequences of preadipocyte differentiation-related genes (PPARγ, C/EBPα, C/EBPβ), lipid metabolism-related genes (FAS, ACC, SREBP1, HSL, ATGL), adipokine genes (FGF21, ZAG) and internal control gene PPIA were listed in the Supplementary Table 1. RT-qPCR was in a final volume of 20 μL and duplicated for two wells. mRNA levels in arbitrary unit were calculated from the value of the threshold cycle (Ct) of the RT-qPCR as related to that of PPIA using the comparative Ct method through formula 2^−ΔΔCT^ (Ct = Ct target gene – Ct mPPIA) (Quesada-López et al., 2016; Yan et al., 2020). Housekeeping gene PPIA was used as internal control to normalize the expression of target genes.

### Western Blot Analysis

3T3-L1 preadipocytes were cultured and fully differentiated as described in *The Effects of Nuciferine on the Differentiation of 3T3-L1 Preadipocytes and Human Primary Preadipocytes*. Fully differentiated adipocytes (day 9) were treated with 0, 2.5, 5, 10 and 20 μM nuciferine for 48h, and then were washed in one x PBS twice and lysed with RIPA lysis buffer supplemented with a protease inhibitor cocktail (Applygen, China). The concentrations of total protein contents were measured followed the instruction of BCA Protein Assay Reagent kit (Beyotime Biotechnology, China). Sodium dodecyl sulfate polyacrylamide gel electrophoresis was used to separate equal protein amounts (30 μg), then samples were electro-transferred onto polyvinylidene difluoride membranes, and blocked with 5% skimmed milk solution and probed with rabbit anti-FGF21 primary antibody (ab171941, Abcam Technology, Beverly, MA, United States, at a dilution of 1:1000). After washed for three times with one x TBST, the membranes were incubated with alkaline phosphatase (AP)-conjugated secondary antibody (goat anti-rabbit IgG-AP, Santa Cruz Biotechnology) for 60 min at room temperature. The signal was amplified by color development using the BeyoECL Plus kit (Beyotime Biotechnology, China) with a stabilized substrate. The bands were then visualized on the Tanon-5200 Chemiluminescent Imaging System (Tanon Science & Technology, Beijing, China) and representative blots are shown. Data is presented as the ratio of the target protein to β-actin.

### Plasmid Transfection and Dual Luciferase Reporter Assay System

pGL3-hFAS (−622∼+3 bp)-Luc (hFAS625-Luc) plasmid was constructed in our laboratory previously (Gong et al., 2006). The verification of the plasmid was displayed in Supplementary Figure 1 3T3-L1 preadipocytes were seeded in 24-well plates with the density of 1 × 10^4^/well. After incubating preadipocytes for 24h, 0.8 μg hFAS625-Luc and 0.2 μg internal control plasmid pRL-SV40 were transiently transfected into 3T3-L1 preadipocytes with 1.5 μL transfection reagent Lipofactamine 3000 (Invitrogen, Los Angles, CA, United States). After 5 h of incubation, the medium was changed to 10% FBS DMEM/F12 solution supplemented with 0, 2.5, 5, 10 and 20 μM nuciferine. 48 h later, the preadipocytes were lysed and both firefly and renilla luciferase activities were measured followed the instruction of the commercial Dual-Luciferase® Reporter Assay System kit (Promega, Madison, WI, United States, Lot 252781) in an automated optical immunoassay analyzer (Beijing Pilot Biotechnology Corporation, China). The firefly luciferase activities were adjusted against the renilla luciferase activities. The experiment was repeated for 3 times, each concentration point was repeated for 4∼5 wells.

### Statistical Analysis

Results were expressed as Mean ± Standard Error (SE). Cell proliferation assay for each experiment was repeated for 3 times, each concentration point and time point were repeated for 9∼12 wells. Cell differentiation, mRNA expression and the promoter activity were repeated for 3 times, and each concentration point were repeated for 4∼5 wells. Chi square test and one-way ANOVA were used for data analysis. The Kruskal-Wallis test was used if the ANOVA was inapplicable. The Dunnett t (two-sided) or Dunnett t3 post-test for three or more groups were used for post hoc test. All statistical computations were run on SPSS 25.0 for Windows (SPSS Inc, Chicago, IL, United States), *p* < 0.05 was considered as statistically significant in all analyses.

## Results

### Nuciferine Inhibited the Proliferation of 3T3-L1 Preadipocytes

**FIGURE 2:**
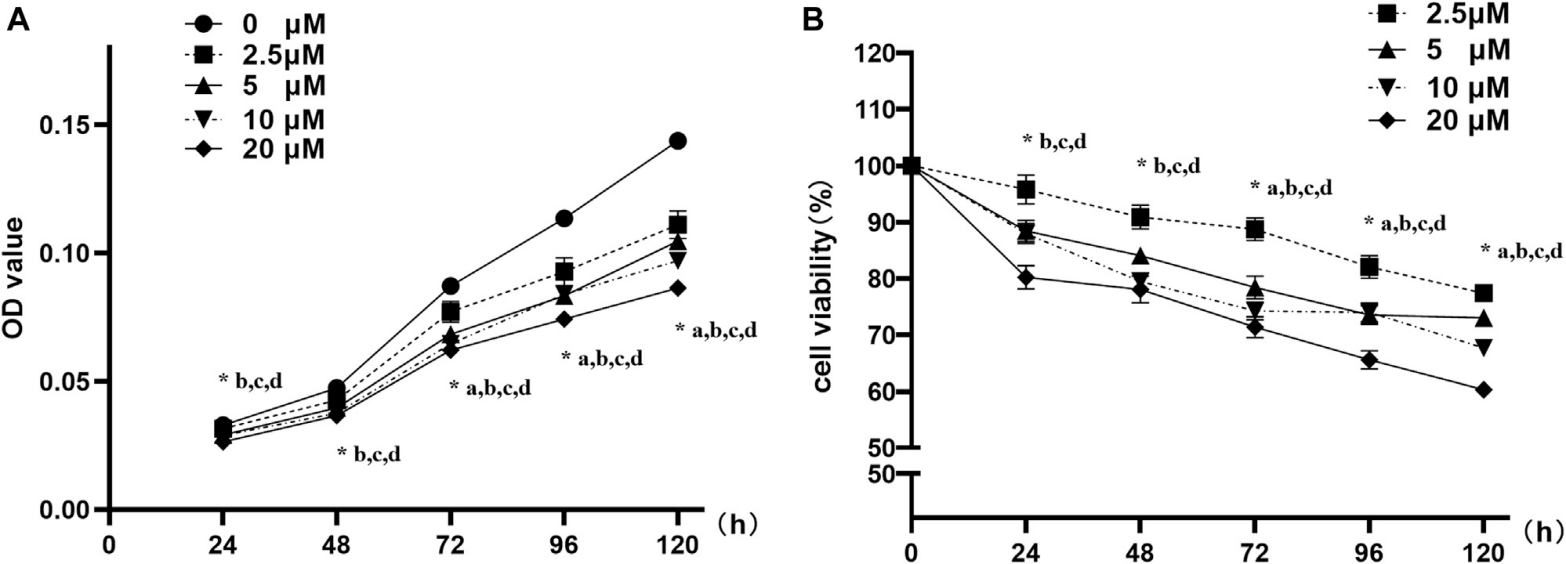
Nuciferine inhibited the proliferation of 3T3-L1 preadipocyte. 3T3-L1 preadipocytes were plated into 96-well plates at a density of 1×10^3^ cells per well. 24 h later, the medium was refreshed and supplemented with different concentrations of nuciferine (0, 2.5, 5, 10 and 20 μM) for different action times (24, 48, 72, 96 and 120 h). Cell proliferation was determined by CCK-8 and the absorbance **(A)** were measured at 450 nm by spectrophotometer. Relative cell viabilities **(B)** were then calculated as the ratio of viable cells against controls (0 μM). Results are presented with mean ± SE from three independent experiments. OD, optical density. **(A)**. *p* < 0.05 2.5 μM vs. the controls (0 μM), **(B)**. *p* < 0.05 5 μM vs the controls (0 μM), **(C)**. *p* < 0.05 10 μM vs the controls (0 μM), **(D)**. *p* < 0.05 20 μM vs the controls (0 μM).

3T3-L1 preadipocytes were treated with 0∼20 μM nuciferine for 24∼120 h. As shown in Figures 2A, 2.5 μM nuciferine significantly reduced OD value by 11.3% compared with the control group (0 μM) at 72 h (*p* < 0.05). As action time extended, the maximal inhibitory effect was observed at 120 h upon 2.5 μM nuciferine administration, where the OD value declined by 22.6% (Figure 2A, *p* < 0.05). With an increase of nuciferine concentrations, the cell growth of 3T3-L1 preadipocytes was suppressed progressively. As shown in Figure 2B, the maximal inhibition effect was observed at 20 μM nuciferine for 120h, where the cell viability rate was 39.7% lower than that of the control cells (0 μM) (Figure 2B, *p* < 0.05). These results indicate that the proliferation of 3T3-L1 preadipocytes was gradually and sustainably inhibited by nuciferine treatment in a dose- and time-dependent manner.

### Nuciferine Decreased Lipid Accumulation and Inhibited Adipogenesis in Differentiating Preadipocytes

#### Nuciferine Decreased Intracellular Lipid Accumulation in Differentiating Preadipocytes

**FIGURE 3:**
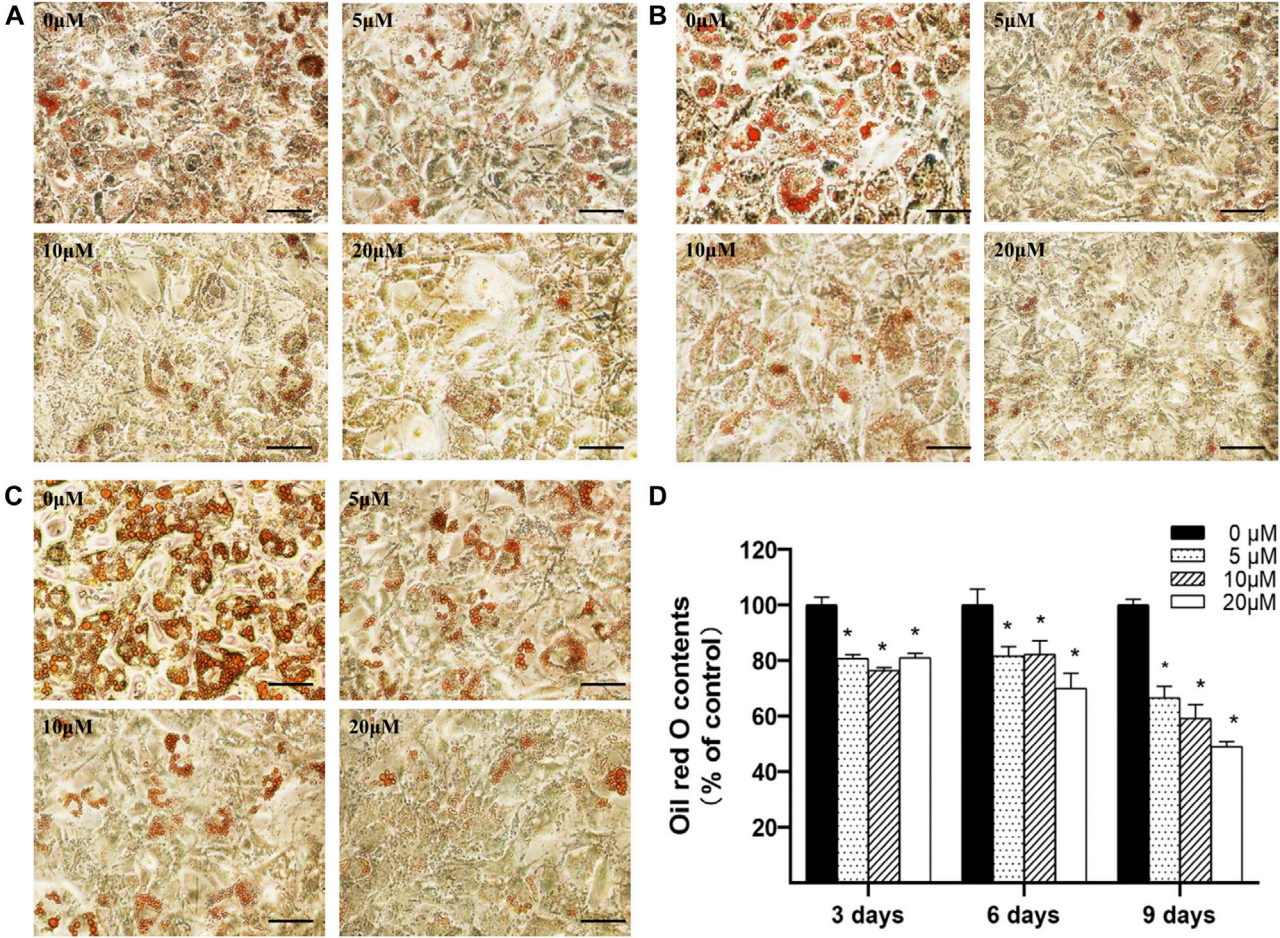
Nuciferine decreased intracellular lipid accumulation in differentiating preadipocytes. 3T3-L1 preadipocytes were differentiated according to the protocol given in the “Methods” section. Preadipocytes were treated with 0, 5, 10 and 20 μM of nuciferine for 3 **(A)**, 6 **(B)** or 9 days **(C)** during the course of differentiation. The cells were photographed at 100x magnification after Oil Red O staining was conducted. Oil Red O dye was then extracted and measured at 492 nm **(D)** using a spectrophotometer. Black scale bar, 200 μm. All results are presented as the mean ± SE from three independent experiments. *p < 0.05 vs the control group (0 μM).

3T3-L1 preadipocytes were seeded into 24-well plates and induced differentiation according to the above protocol. As the differentiation process initiated, the cells were cultured with differentiation medium supplemented with 0∼20 μM nuciferine for 3, 6 and 9 days, and cells were then stained by Oil O Red dye and photographed on each experiment time point. As depicted in Figure 3A, after 3 days of differentiation, lipid droplets in untreated control group were stained with Oil red O, while in cells treated with 5∼20 μM nuciferine, the number and size of stained lipid droplets have progressively decreased as the nuciferine concentrations increased. With the extension of action time, the inhibitory effect of nuciferine on lipid contents enhanced (Figures 3B,C). The quantitative analysis revealed that the lipid contents in differentiating cells was gradually and remarkably reduced upon nuciferine treatment (Figure 3D). The maximal reduction effect was observed after cells were treated with 20 μM nuciferine for 9 days, as the lipid contents was declined by 50.9% in comparison with the control group (0 μM) (*p* < 0.05).

#### Nuciferine Decreased Intracellular Triglyceride Contents in Differentiating Preadipocytes

**FIGURE 4:**
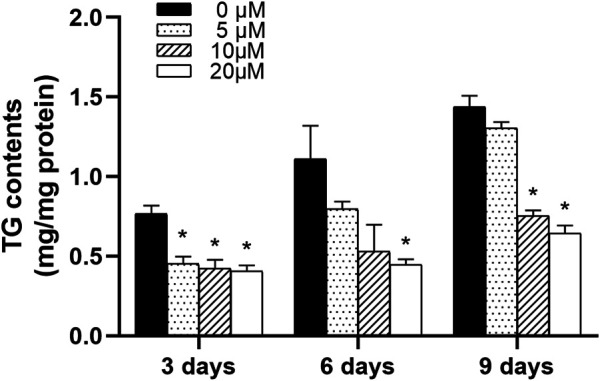
Nuciferine decreased intracellular TG contents in differentiating preadipocytes. 3T3-L1 preadipocytes were induced differentiation following the differentiation protocol described in the “Method” section. Preadipocytes were treated with nuciferine at different concentrations (0, 5, 10, 20 μM) for various action times (3, 6, 9 days) during the course of differentiation. Intracellular TG contents was adjusted against total protein contents. All results were presented as mean ± SE from three independent experiments. *p < 0.05 vs the control group (0 μM).

3T3-L1 preadipocytes were differentiated and treated with 0∼20 μM nuciferine for 3, 6 and 9 days. Cells were then harvested to determine the intracellular TG contents. As showed in Figure 4, after treated for 3 days, 5 μM nuciferine significantly decreased the intracellular TG contents in differentiating preadipocytes. The inhibitory effect was reinforced with the increase in nuciferine concentration. As in cells treated with 20 μM nuciferine for 3 days, the lipid contents was decreased by 47.2% when compared with the control group (0 μM) (*p* < 0.05). And in line with this result, 20 μM nuciferine remarkably reduced the accumulation of intracellular TG contents after 6 and 9 days of treatment, reducing by 59.9 and 55.4% as compared with the controls (0 μM), respectively (Figure 4, *p* < 0.05).

#### Nuciferine Inhibited the Expression of Differentiation- and Lipid-Metabolism-Related Genes in Differentiating Preadipocytes

**FIGURE 5:**
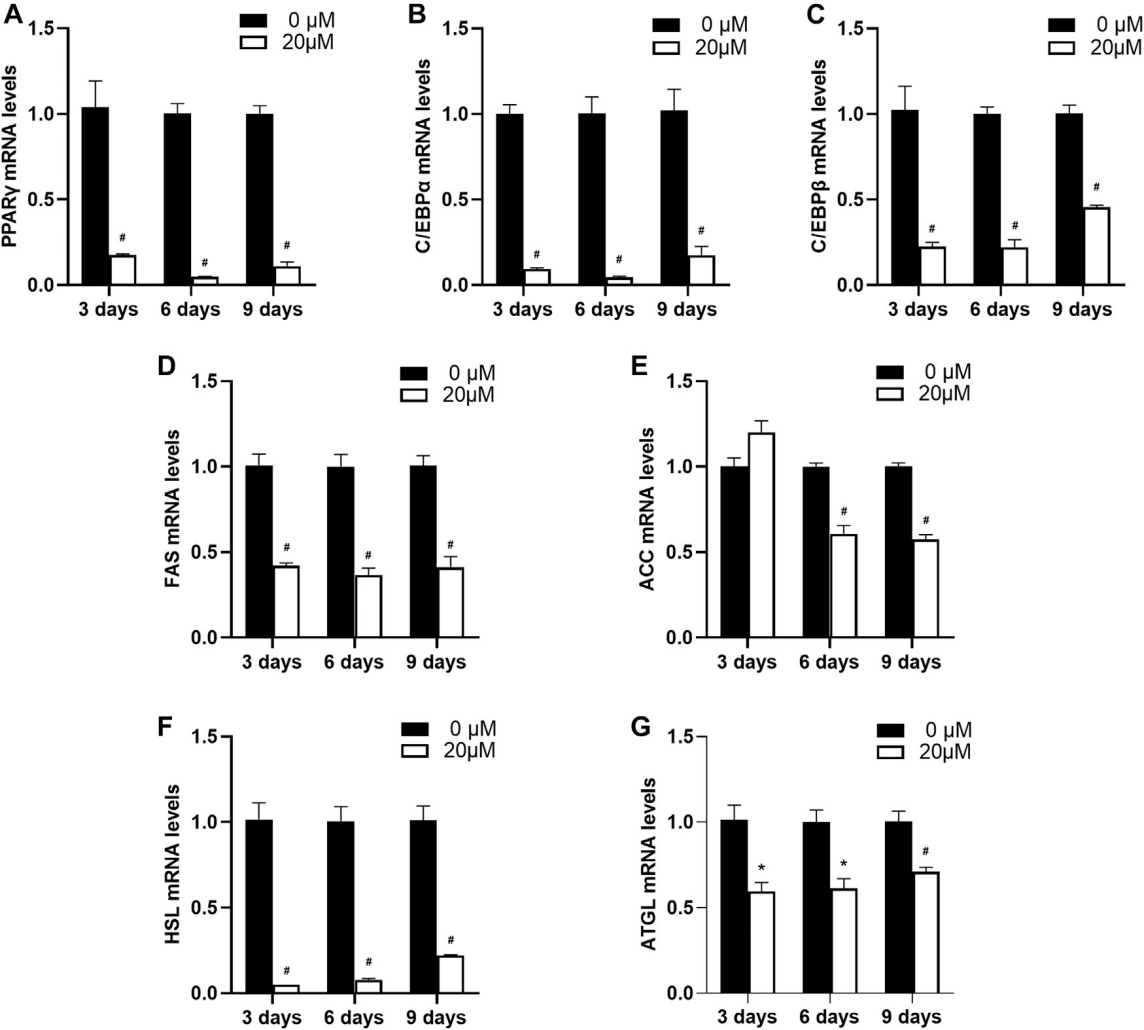
Nuciferine inhibited the expression of differentiation- and lipid-metabolism-related genes in differentiating preadipocytes. 3T3-L1 preadipocytes were treated with or without 20 μM nuciferine for 3,6 and 9 days during the course of differtiation. The total RNA were extrated on each time points and the mRNA levels of PPARγ **(A)** and C/EBPα **(B)**, C/EBPβ (**C**), FAS (**D**), ACC (**E**), HSL (**F**) and ATGL (**G**) in 3T3-L1 cells were determined by RT-qPCR. All of the results were normalized to the values of the internal control PPIA, and the results were expressed as fold changes of the Ct value relative to the control value, which was defined as 1. All results were presented as mean ± SE from three independent experiments. #*p* < 0.001 vs. the controls (0 μM), *p < 0.05 vs. the controls (0 μM).

Next, as 20 μM nuciferine was shown to possess a potent inhibitory effect on intracelluar lipid accumulation in differentiating preadipocytes, we anaylzed the expression of differentiation- and lipid-metabolism-related genes on equal conditions. As shown in Figure 5, the relative mRNA levels of PPARγ, C/EBPα and C/EBPβ were remarkably suppressed. The maximal reduction was obeserved after 6 days of treatment where the mRNA levels of PPARγ, C/EBPα and C/EBPβ exhibited a sharp decline of 95.3%, 95.5 and 78.0% compared with the control group (0 μM), respectively (Figures 5A–C *p* < 0.001). Moreover, the expressions of gene related to adipogenesis including FAS and ACC were also decreased. After treated with 20 μM nuciferine for 3 days, the mRNA levels of FAS were significantly reduced by 58.0% compared with the control group (0 μM), and the maximum inhibition effect was observed after 6 days, as the FAS mRNA levels were decreased by 63.5% as compared with the controls (0 μM) (Figure 5D, all *p* < 0.001). The mRNA levels of ACC were notablely decreased by 39.2 and 42.5% compared with the controls (0 μM) upon 6 and 9 days treatment of nuciferine, respectively (Figure 5E, *p* < 0.001). As for lipolysis-related gene HSL, the result showed that nuciferine significantly lowered the mRNA levels of which by 95.1, 92.3 and 78.2% in comparasion with the controls (0 μM) after being treated with nuciferine for 3, 6 and 9 days, respectively (Figure 5F, all *p* < 0.001). Besides, The mRNA levels of ATGL were also significantly deceased upon nuciferine treatment (Figure 5G, *p* < 0.05).

### Nuciferine Inhibited Lipogenesis and Promoted the Expression of Adipokines in Fully Differentiated Adipocytes

#### Nuciferine at Different Concentrations Inhibited the Expression of Lipogenesis-Related Genes in Fully Differentiated Adipocytes

**FIGURE 6:**
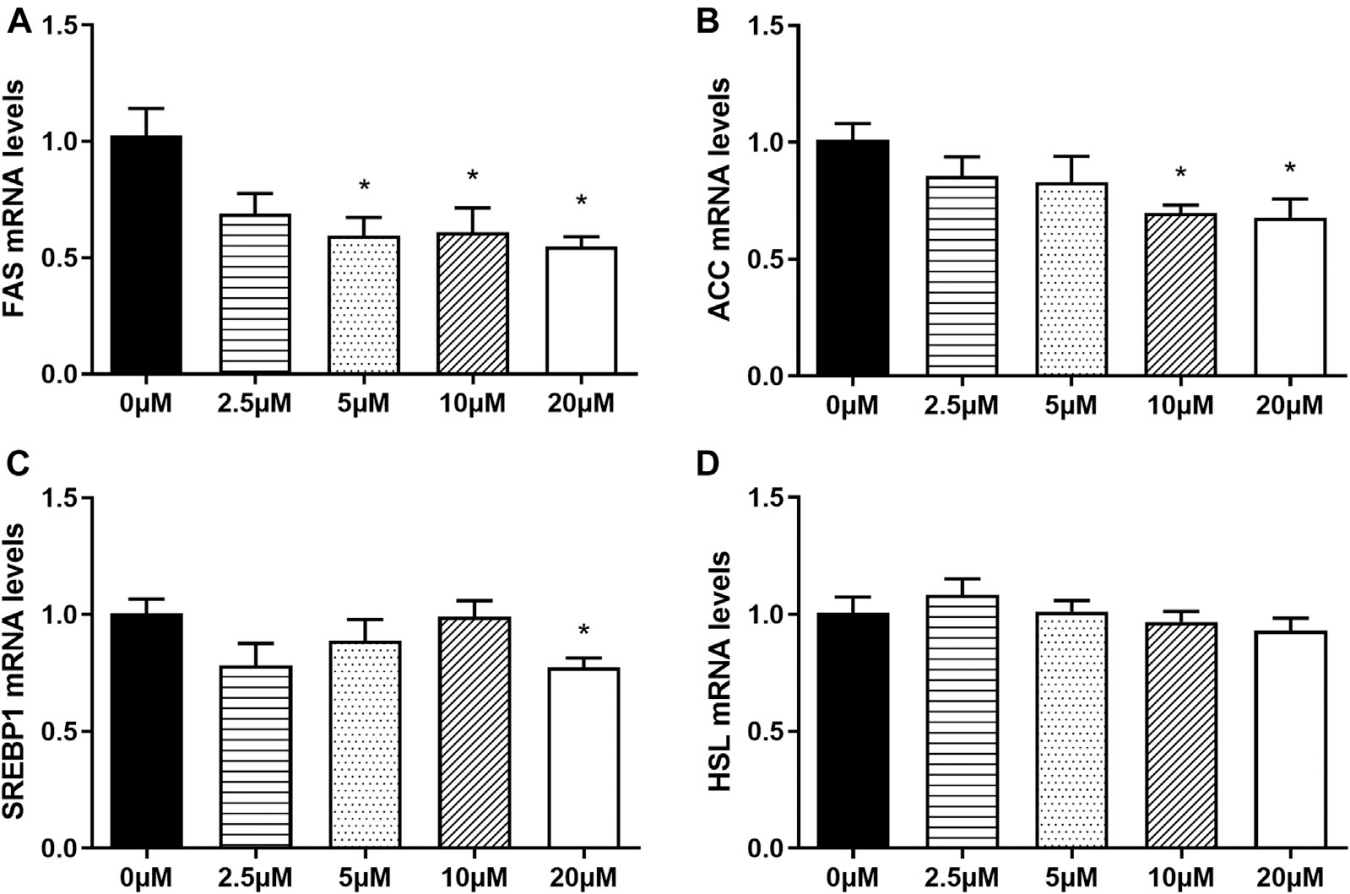
Nuciferine at different concentrations inhibited the expression of lipogenesis-related genes in fully differentiated adipocytes. 3T3-L1 preadipocytes were differentiated as described in “Method” section. On the ninth day following differentiation, the fully differentiated adipocytes were treated with 0, 2.5, 5, 10, 20 μM nuciferine for 48 h, then the total RNA was extracted and RT-qPCR were conducted to determine the mRNA levels of FAS **(A)**, ACC **(B)**, SREBP1 **(C)** and HSL **(D)**. All of the results were normalized to the values of the internal control PPIA, and the results were expressed as fold changes of the Ct value relative to the control value, which was defined as 1. All results were presented as mean ± SE from three independent experiments. *p < 0.05 vs the control group (0 μM).

In order to explore whether nuciferine affect the expression of lipogenesis-related genes in mature adipocytes, the fully differentiated adipocytes (day 9) were treated with 0∼20 μM nuciferine for 48 h, and the expression of genes related to lipid metabolism were determined subsequently. As depicted in Figure 6, 5 μM nuciferine reduced the mRNA level of FAS by 40.4% when compared with the control group (0 μM), while the maximal suppression action was induced by 20 μM nuciferine, presenting a reduction 45.2% lower than the controls (0 μM) (Figure 6A, *p* < 0.05). Meanwhile, the mRNA levels of ACC in cells treated with 10 and 20 μM nuciferine were lowered by 30.2 and 32.2%, respectively, compared with the control group (0 μM) (Figure 6B, *p* < 0.05). As for SREBP1, the expression was also significantly decreased by 22.6% in comparison of the controls (0 μM) after treated with 20 μM nuciferine (Figure 6C, *p* < 0.05). However, there was no notable difference observed in HSL mRNA levels in fully differentiated adipocytes treated with or without nuciferine (Figure 6D).

#### Nuciferine Inhibited the Expression of Lipogenesis-Related Genes in Fully Differentiated Adipocytes at Different Action Times

**FIGURE 7:**
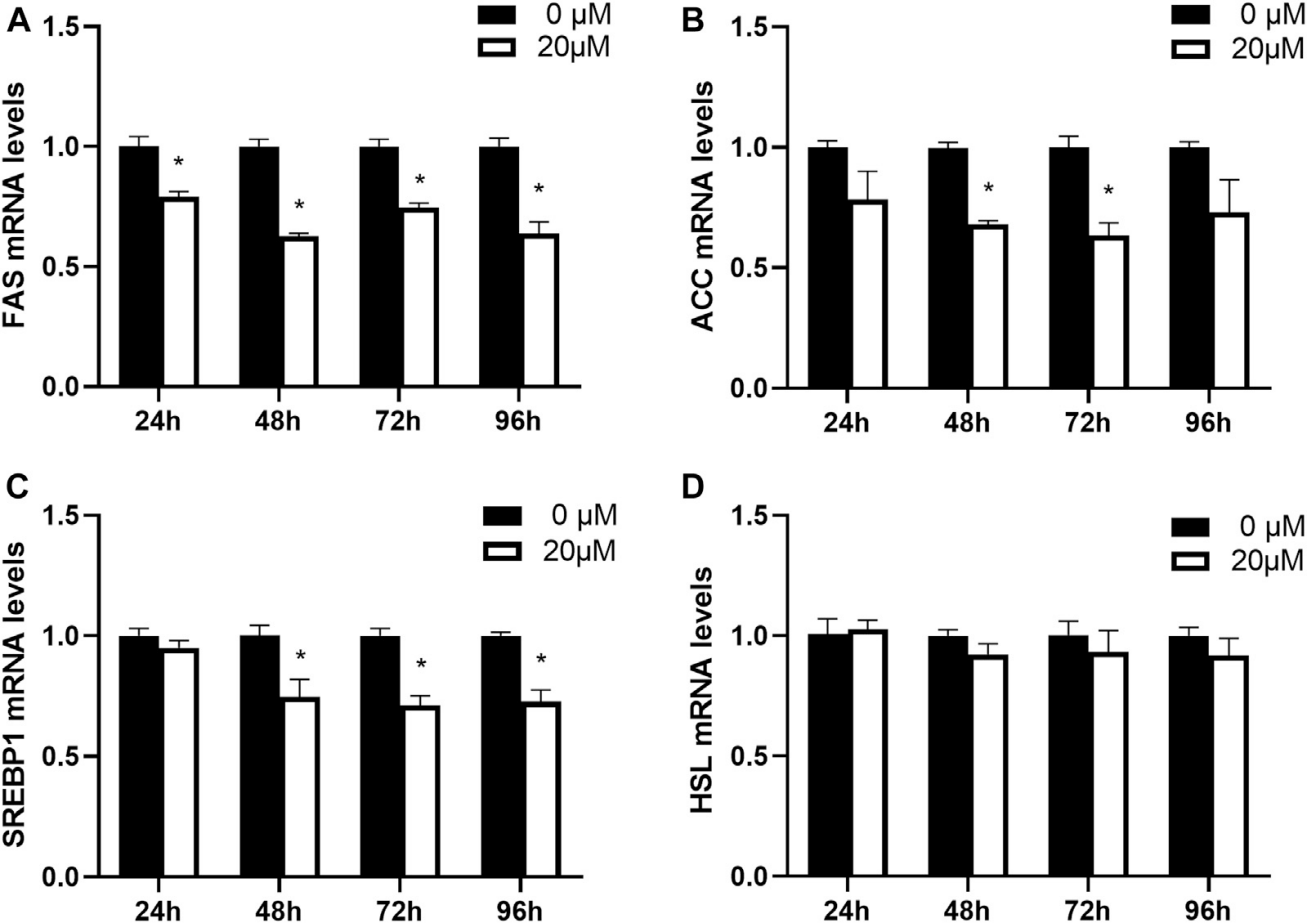
Nuciferine inhibited the expression of lipogenesis-related genes in fully differentiated adipocytes at different action times. 3T3-L1 preadipocytes were differentiated as described in “Method” section. On the ninth day following differentiation, the fully differentiated adipocytes were treated with 20 μM nuciferine for 24, 48, 72 and 96 h, then the total RNA was extracted and RT-qPCR were conducted to determine the mRNA levels of FAS **(A)**, ACC **(B)**, SREBP1 **(C)** and HSL **(D)**. All of the results were normalized to the values of the internal control PPIA, and the results were expressed as fold changes of the Ct value relative to the control value, which was defined as 1. All results were presented as mean ± SE in three independent experiments. *p < 0.05 vs the control group (0 μM).

Next, the effects of nuciferine on the expression of lipogenesis-related genes in fully differentiated adipocytes at different action times were investigated. The fully differentiated adipocytes were treated with 20 μM nuciferine for 24, 48, 72 and 96 h. On each time point, cells were lysed and the expression of related genes were measured. As presented in Figure 7, the relative mRNA level of FAS was notably decreased in cells treated with 20 μM nuciferine for 24 h. The maximal reduction action was found after 48 h, where the mRNA leves of FAS was reduced by 37.4% when compard with the control group (0 μM) (Figure 7A, *p* < 0.05). In consistence with the results of ACC mRNA levels obtained in the experiments conducted with nuciferine at different concentrations, 48 h of nuciferine adiministration also yielded a significant reduction of 32.0% on ACC mRNA levels, and the reduction rate slightly increased at 72 h, reaching 36.7% when compared with the controls (0 μM) (Figure 7B, *p* < 0.05). Moreover, nuciferine also significantly decreased the mRNA levels of SREBP1 by 25.3% for 48 h of treatment when compared with the controls (0 μM), and fell off by 29.0 and 27.3% after treated with nuciferine for 72h and 96 h (Figure 7C, *p* < 0.05). However, there was still no significant difference on HSL mRNA levels between groups treated with or without nuciferine at different action times, similar to the results obtained when cells were treated with nuciferine at different concentrations (Figure 7D).

#### Nuciferine Promoted the Expression of Adipokines in Fully Differentiated Adipocytes

**FIGURE 8:**
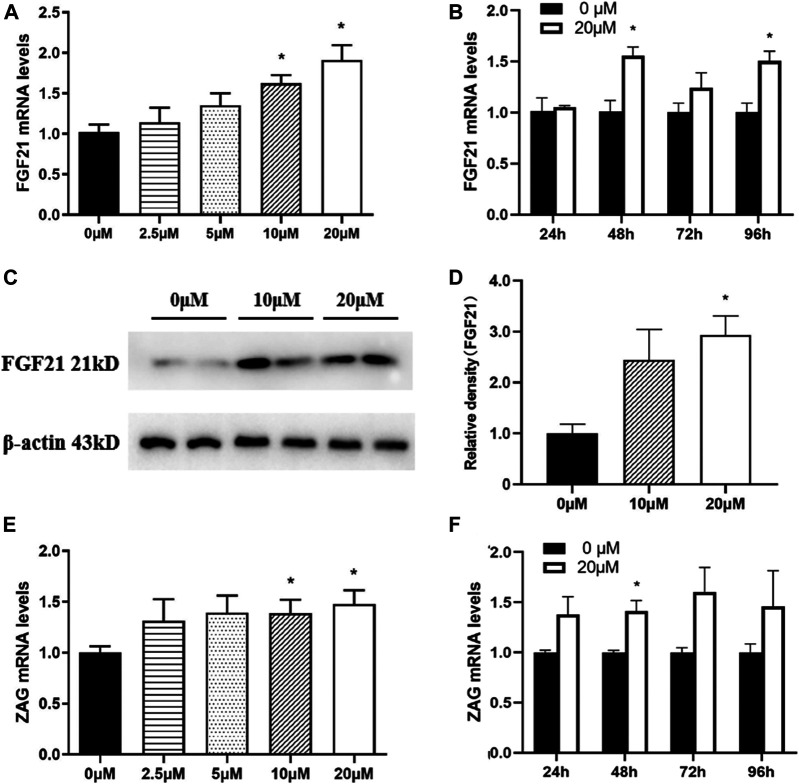
Nuciferine promoted the expression of adipokines in fully differentiated adipocytes. 3T3-L1 preadipocytes were differentiated as described in the “Methods” section. On the ninth day of differentiation, the fully differentiated adipocytes were exposed to either 0, 2.5, 5, 10, 20 μM nuciferine for 48 h or 20 μM nuciferine for 24, 48, 72 and 96 h. Then the total RNA was extracted and RT-qPCR were conducted to determine the mRNA levels of FGF21 **(A, B)** and ZAG **(E, F)** in cells undergone different conditions. The protein levels of FGF21 in fully differentiated cells treated with nuciferine at different concentration were determined by western blot analysis **(C)**, and the density ratio of FGF21 was calculated using β-actin as the control **(D)**. All results are presented with mean ± SE from three different experiments, *p < 0.05 compared with the control group (0 μM).

FGF21 and ZAG are two key adipokines which embrace a plethora of metabolic functions throughout the body, especially in adipose tissue. As shown in Figure 8A, an increase in nuciferine concentrations gradually promoted the mRNA levels of FGF21 in fully differentiated adipocytes. The mRNA level of FGF21 in cells treated with 20 μM nuciferine for 48 h was 1.91 folds higher than the control group (0 μM) (Figure 8A, *p* < 0.05). At the same time, the protein levels of FGF21 also significantly increased to 2.93 folds higher than the controls (0 μM) (Figures 8C,D, *p* < 0.05). Similar results were obtained when cells were treated with nuciferine at different action times, with the maximal stimulatory effect observed after 48 h of treatment, where the mRNA levels of FGF21 was 1.56 folds higher in comparison with the control group (0 μM) (Figure 8B, *p* < 0.05). Besides, as shown in Figure 8E, the mRNA levels of ZAG were significantly promoted by 10 μM nuciferine and the increments further augmented with increasing concentrations of nuciferine. The maximal stimulatory action was noted to be 1.48 folds above the controls (0 μM) after treated fully differentiated adipocytes with 20 μM nuciferine (Figure 8E, *p* < 0.05). When adipocytes were treated by nuciferine at different action times, it was observed that 48 h treatment of nuciferine significantly increased the mRNA levels of ZAG, where it was 1.41 folds higher than that of the controls (0 μM) (Figure 8F, *p* < 0.05).

### Nuciferine Inhibited FAS Promoter Activities in 3T3-L1 Preadipocytes

**FIGURE 9:**
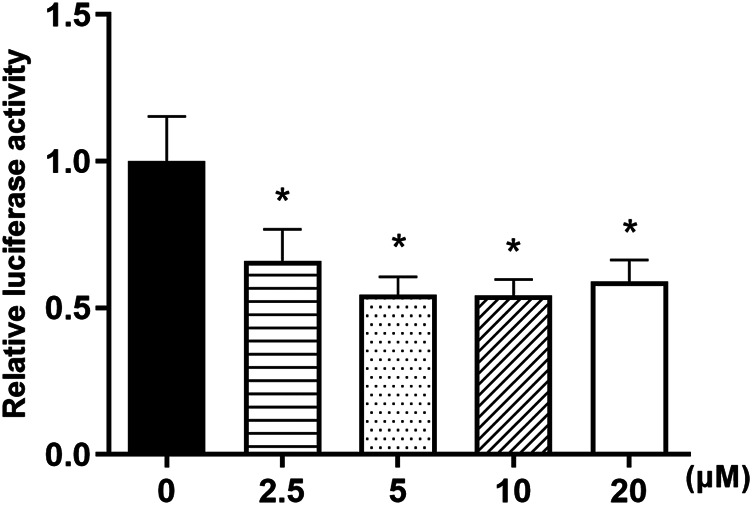
Nuciferine inhibited FAS promoter activities in 3T3-L1 preadipocytes. The 3T3-L1 preadipocytes were plated into 24-wells plates at a density of 1×10^4^/well and cultured for 24 h, then these cells were transiently transfected with a luciferase reporter gene plasmid hFAS625-Luc and an internal control plasmid pRL-SV40 using Lipofectamine 3000. 5 h later, the cells were treated with different concentrations of nuciferine (0, 2.5, 5, 10 and 20 μM) for 48 h. The firefly and renilla luciferase activities were then measured in cell lysis. The firefly luciferase activities were adjusted by the renilla luciferase activities. All results are presented with mean ± SE from three independent experiments. *p < 0.05 vs the control group (0 μM).

Owing to our findings above which indicated that nuciferine may have strong and sustainable inhibiting effect on the mRNA levels of FAS, plasmids containing human FAS promoter were used to investigate the effect of nuciferine on FAS promoter activities. 3T3-L1 preadipocytes were transiently transfected with hFAS-625-luc plasmids and treated with nuciferine at different concentrations for 48 h. As shown in Figure 9, 2.5 μM nuciferine significantly suppressed the luciferase activities in 3T3-L1 preadipocytes. The maximum reduction concentration was observed at 10 μM, where the luciferase activities dropped 45.8% lower compared with the control groups (0 μM) (*p* < 0.05). It was observed that in preadipocytes treated with 5 and 20 μM nuciferine, the luciferase activities were declined by 45.6 and 41.0% of the control groups (0 μM), respectively (Figure 9, *p* < 0.05).

### Nuciferine at Different Concentrations Regulated the Expression of Lipid-Metabolism-Related Genes and Promoted the Expression of Adipokines in Fully Differentiated Human Primary Adipocytes

**FIGURE 10:**
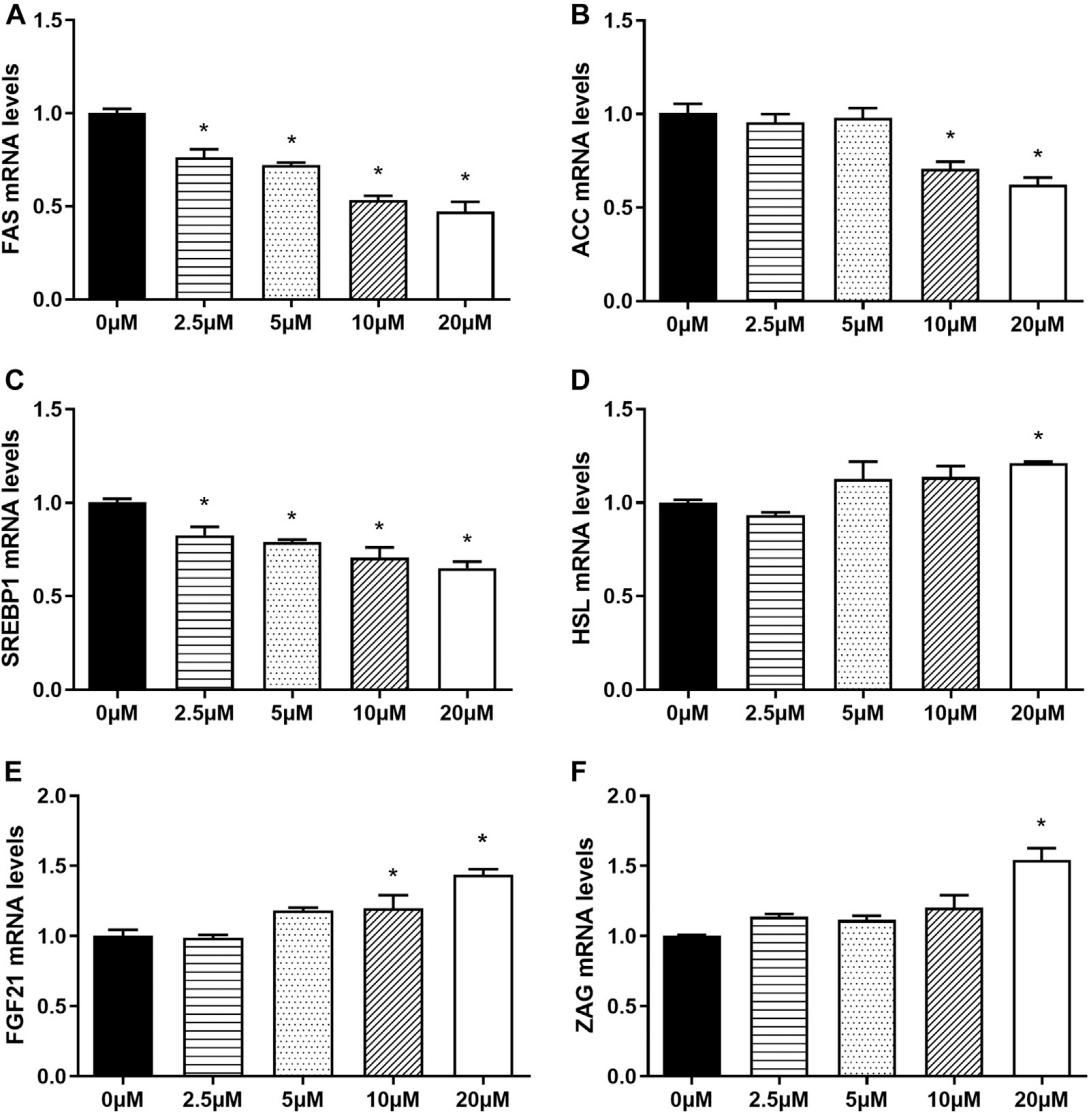
Nuciferine regulated the expression of lipid-metabolism-related genes and promoted the expression of adipokines in fully differentiated human primary adipocytes. The human primary preadipocytes were plated into 24-wells plates at a density of 2 × 10^4^/well, 48 h after complete confluence, the cells were differentiated as described in the “Methods” section. On the ninth day of differentiation, the fully differentiated adipocytes were exposed to 0, 2.5, 5, 10, 20 μM nuciferine for 48 h. Then the total RNA was extracted and RT-qPCR were conducted to determine the mRNA levels of FAS **(A)**, ACC **(B)**, SREBP1 **(C)** HSL **(D)**, FGF21 **(E)** and ZAG **(F)**. All of the results were normalized to the values of the internal control PPIA, and the results were expressed as fold changes of the Ct value relative to the control value, which was defined as 1. All results were presented as mean ± SE in three independent experiments. *p < 0.05 vs the control group (0 μM).

In order to further investigate the effects of nuciferine on the expression of lipogenesis-related genes and adipokines, human primary preadipocytes were induced differentiation and then treated with different concentrations of nuciferine (0, 2.5, 5, 10, 20 μM) for 48 h. As shown in Figure 10A, 2.5 μM nuciferine significantly decreased the mRNA levels of FAS by 24.7% (*p* < 0.05). With concentrations raised, the inhibitory effect of nuciferine augmented. 20 μM nuciferine notably inhibited the mRNA levels of FAS by 52.7% (Figure 10A, *p* < 0.05). Similarly, 20 μM nuciferine significantly decreased the mRNA levels of ACC and SREBP1 by 37.7 and 35.0%, respectively (Figures 10B,C, *p* < 0.05). Interestingly, it was found that in human primary adipocytes, 20 μM nuciferine significantly enhanced the expression of HSL, which was up to 121.0% compared with the control group (0 μM) (Figure 10D, *p* < 0.05). Moreover, in consistent with the observation in 3T3-L1 adipocytes, the mRNA levels of adipokines FGF21 and ZAG were elevated upon nuciferine treatment. The maximal promotion effect was observed at 20 μM, where the mRNA levels of FGF21 and ZAG was up to 143.7 and 154.3% compared with the control group (0 μM), respectively (Figures 10E,F, *p* < 0.05).

## Discussion

In Chinese traditional medicine, lotus leaf (also known as *Nelumbo nucifera* leaf) has been used as a medicine for cardiovascular diseases, type 2 diabetes and tumors. Recent studies have revealed that the pharmacological effects of lotus leaf are closely related to its main aporphine alkaloid component, nuciferine. It was reported *in vivo* that nuciferine improved metabolic profile in obese mice (Nguyen et al., 2012; Zhang et al., 2018). In our present study, we found that *in vitro*, nuciferine could inhibit the proliferation and differentiation of 3T3-L1 preadipocytes and reduce lipid accumulation during differentiation by inhibiting the expression of differentiation-related transcription factors and lipid metabolism-related enzymes. Similar results were obtained in human primary preadipocytes. Further experiments demonstrated that nuciferine suppressed lipogenesis, at least partly, through decreasing FAS mRNA expression by repressing FAS promoter activities. Besides, nuciferine promoted the expression of FGF21 and ZAG in fully differentiated adipocytes and human primary adipocytes, which was known to have beneficial actions when activated (Fisher and Maratos-Flier, 2016; Wei et al., 2019).

In this study, it was found that nuciferine could gradually inhibit the proliferation of 3T3-L1 preadipocytes with concentration (0∼20 μM) and action time (0∼120 h) extended, suggesting that nuciferine is capable of inhibiting the proliferation of 3T3-L1 preadipocytes in a dose- and time-dependent manner. In consistence with our results, several studies showed that nuciferine possessed the property of inhibiting the proliferation of various tumor cells, including neuroblastoma cells, colorectal cancer cells, breast cancer cells and glioblastoma cells (Qi et al., 2016; Kang et al., 2017; Li et al., 2019). (Qi et al., 2016) showed that nuciferine (0.8 mg/ml) suppressed the proliferation of human neuroblastoma SY5Y cells and murine colorectal cancer CT26 cells by inhibiting the PI3K/AKT signaling pathway. Similarly, (Kang et al., 2017) found that 60 μM nuciferine could induce G1 phase arrest of breast cancer cell line MDA-MB-231 hence inhibiting cell proliferation. (Li et al., 2019) also found that 60 μM nuciferine could down-regulate the expression of G2 phase marker protein CDC2 in glioma cells, indicating that nuciferine could inhibit cell proliferation by reducing the number of cells entering G2/M phase. Besides its effect on cell proliferation, recent studies have shown that nuciferine decreased the ratio of two apoptosis-related genes, Bcl-2/Bax, thus leading to cell apoptosis in human lung adenocarcinoma A549 cells (Liu et al., 2015).

In the presence of a mixture of insulin, dexamethasone and IBMX, 3T3-L1 preadipocytes can be induced to differentiation. As differentiation initiates, cytoplasmic lipid accumulation gradually occurred with time. In our present study, we found for the first time that the treatment of the preadipocytes with nuciferine for 3 days following differentiation, which can be referred as the early phase of differentiation, significantly reduced the intracellular lipid accumulation and TG contents, implying that nuciferine suppressed early adipogenesis in differentiating 3T3-L1 cells. As the action time of nuciferine extended, the inhibitory effect of nuciferine on intracellular lipid accumulation persisted until the late phase of differentiation (9 days), further elucidating that nuciferine had the capacity of inhibiting lipid accumulation not only during early onset but throughout the whole process of differentiation. Similarly (Siegner et al., 2010), cultivated preadipocytes in differentiation medium supplemented with 0.5% or 1% lotus leaf extract solution for 7 days, and found that 0.5% lotus leaf extract solution significantly reduced TG levels to 77 ± 3.4% of the controls, while 0.1% lotus leaf extract solution notably decreased TG levels to 46 ± 5.9%. (Ahn et al., 2013) also found that alkaloid compound extracted from lotus leaves could significantly inhibit adipocyte differentiation and reduce lipid accumulation. As nuciferine being the main bioactive component of lotus leaf extract, researchers took a step further exploring the effect of nuciferine in 3T3-L1 preadipocytes. (Ma et al., 2015), found that 2 mg/L nuciferine decreased the lipid droplets and the intracellular triglyceride contents but increased the glucose uptake in the insulin resistant mature 3T3-L1 adipocytes. (Yoo et al., 2019) induced differentiation of 3T3-L1 preadipocytes in presence with nuciferine, and found that nuciferine significantly reduced adipogenesis in 3T3-L1 cells. Similar results were obtained by (Liu et al., 2020), who found that 0∼100 μg/ml nuciferine significantly decreased TG and TC contents in differentiating 3T3-L1 preadipocytes. These studies, together with ours, indicated that nuciferine might be the very component that possesses the predominant effect of inhibiting intracellular lipid accumulation during the process of preadipocyte differentiation.

Differentiation of preadipocytes is a complex biochemical process regulated by series of factors. Among which C/EBPβ is a crucial adipogenic transcription factor induced initially upon differentiation stimuli, then C/EBPα and PPARγ were induced to cooperatively promote adipogenesis alongside the process of differentiation (Lefterova et al., 2008). In the present study, the mRNA levels of C/EBP β, PPARγ and C/EBPα were found to be significantly deceased on the third, sixth and ninth day of differentiation upon nuciferine treatment. Similarly (Yoo et al., 2019), found that 8 days of nuciferine supplementation in differentiation medium of 3T3-L1 preadipocytes significantly reduced adipogenesis and the expression of adipogenic transcription factors including PPARγ and C/EBPα. In accordance with this findings, our previous studies found that 6 weeks intervention of lotus leaf aqueous extract notably reduce fat mass and suppressed mRNA levels of PPARγ in visceral adipose tissue of HFD induced obese rats (Yan et al., 2017). Feng S demonstrated that the inhibition of C/EBP β, PPARγ and C/EBPα in adipose tissue plays an important role in blocking the differentiation of preadipocytes and inhibiting intracellular lipid accumulation (Feng et al., 2016). Taken together, these results indicate that nuciferine may reduce the amount of adipose tissue via suppressing preadipocyte differentiation by inhibiting the expression of differentiation-related transcription factors. In addition, we found that in differentiating preadipocytes, the mRNA levels of FAS and ACC, two key enzymes of fat synthesis, were decreased by the treatment of nuciferine. Interestingly, the mRNA levels of HSL and ATGL, which are the major enzymes in adipose tissue contributing to the catabolism of TG, were also decreased. Since it is widely acknowledged that PPARγ is a crucial regulator on the balance between fat synthesis and lipolysis, studies have demonstrated the defective PPARγ is associated with the decreased expression of lipolysis-related genes including ATGL and HSL *in vivo* and *in vitro* (Rodriguez-Cuenca et al., 2012). Moreover, the degradation of PPARγ was reported to reduce mRNA levels of ATGL and HSL in the differentiated adipocytes (Jin et al., 2014). It is assumed that the decrease of HSL and ATGL mRNA levels in the differentiating preadipocytes in our present study were consequent to the downregulation of PPARγ induced by nuciferine. Taken together, these results suggested that nuciferine could inhibit both fat synthesis and lipolysis during differentiation, but its inhibitory effect on fat synthesis dominated and led to the final suppression effect of the lipid accumulation in the differentiating preadipocytes.

It is well known that the intracellular lipid contents of mature adipocytes are determined by the balance between lipogenesis and lipolysis. Studies have shown that several key transcription factors and enzymes such as SREBP1, FAS, ACC and HSL are involved in the regulation of fat synsthesis and breakdown. In the present study, it was found that in fully differentiated adipocytes, nuciferine decreased the mRNA levels of transcription regulator SREBP1 and its downstream enzymes FAS, ACC at 5∼20 μM for 48 h. A study performed by (Yoo et al., 2019) supported our findings, reporting in 3T3-L1 preadipcocytes, nuciferine supplementation in differentiation medium reduced lipid accumulation as well as the protein level of SREBP1 and FAS during differentiation through Akt-mTORC1 signaling pathway. Moreover, in our study, similar effect was observed in fully differentiated human primary adipocytes. Siegner R et al. also found in primary cultured human adipocytes that lotus leaf extract reduced the contents of intracellular lipid and inhibited the expression of SREBP1 (Siegner et al., 2010). Consistently, Guo F et al. found that 10 mg/kg nuciferine supplementation significantly reduced the hepatic mRNA expression of SREBP1, FAS and ACC and rescued the adverse metabolic changes brought by HFD diet in hamsters (Guo et al., 2013). These results together indicated that the beneficial effects of nuciferine supplementation may attributed partly to its ability of suppressing the expressions of SREBP1 and its downstream enzymes.

FAS is a key lipogenesis enzyme which decarboxylates malonyl-Coenzyme A (CoA) and acetyl-CoA into fatty acids. Fan et al. (Fan et al., 2013) found that the inhibition of FAS in adipocytes could reduce intracellular lipid contents, and similar results were obtained by (Habinowski and Witters, 2001) who found that the differentiation of 3T3-L1 preadipocytes was inhibited when the expression of FAS was blocked. In accordance, in our present study, we found that nuciferine significantly inhibited the mRNA levels of FAS in differentiating and fully differentiated adipocytes, suggesting that the inhibitory effect of nuciferine on lipid accumulation is related to the downregulation of FAS. Furthermore, the results obtained with the transient hFAS 625-Luc plasmid transfection experiment indicated that nuciferine significantly inhibited the luciferase activities of FAS promoter in 3T3-L1 preadipocytes, implying that nuciferine may suppress FAS expressionby directly reducing FAS promoter activities.

Adipokines are a number of proteins synthesized and secreted mainly by adipocytes. Among them, FGF21 was reported to possess the properties of promoting the oxidation of fatty acids and inhibiting adipogenesis, thus play an important role in metabolic regulation of the whole body (Tezze et al., 2019). In this study, we found that 48 h treatment of 20 μM nuciferine could significantly promote the mRNA and protein levels of FGF21 in the fully differentiated adipocytes, suggesting that the inhibitory effect of nuciferine on lipogenesis in adipocytes may also achieved through promoting the beneficial adipokine FGF21 expression in addition to its direct inhibitory effect of the expression of key lipogenesis enzymes in 3T3-L1 adipocytes. One possible explanation for this phenomenon is based on studies conducted by Salminen A et al. who showed that FGF21 could activate AMPK pathway (Salminen et al., 2017), while (Zhang et al., 2020) found that activation of AMPK pathway could inhibit the expression of SREBP1, FAS and ACC. Therefore, we raised a presumption that nuciferine may promoted the expression of FGF21 and subsequently activate intracellular AMPK pathway, thus inhibiting the expression of enzymes related to fat synthesis and reducing intracellular fat contents in 3T3-L1 adipocytes. In addition, we also found that 48 h treatment of 10∼20 μM nuciferine could significantly increase the mRNA levels of ZAG, another adipokines reported to be of great importance in body weight regulation (Wang et al., 2020). Several studies together with our previous studies showed that the levels of ZAG in serum and adipose tissue of obese patients are significantly lower than those of normal weight subjects (Selva et al., 2009; Liu et al., 2020). *In vivo*, it was found that ZAG induced body weight loss and fat mass reduction (Gong et al., 2010; Liu et al., 2018; Gong et al., 2009). To elaborate, the administration of ZAG in obese rodents could significantly reduce body weight and fat mass by decreasing the expression of FAS and ACC in liver and adipose tissue (Gong et al., 2010; Russell and Tisdale, 2011). Besides, it is also worth noting that our previous study also found that ZAG can directly inhibit the differentiation and lipid accumulation of 3T3-L1 preadipocytes (Zhu et al., 2013). Taken together, the resuls indicate that nuciferine may inhibit lipid accumulation through promoting the expression of beneficial adipokine ZAG. We also measured the mRNA levels of FGF21 and ZAG in human primary adipocytes upon nuciferine treatment and similar effect was observed. All of these findings indicated that nuciferine inhibits the lipid accumulation of 3T3-L1 adipocytes and human primary adipocytes, on the one hand, by inhibiting the expression of key lipogenesis enzymes, on the other hand, by inhibiting the expression of adipokines, which in turn further inhibits fat synthesis in the way of autocrine.

In conclusion, it was found that nuciferine inhibited the proliferation of 3T3-L1 preadipocytes in a dose- and time-dependent manner. Moreover, Nuciferine suppressed the differentiation of 3T3-L1 cells by inhibiting the expression of PPARγ and C/EBPα, and it was also capable of reducing lipid accumulation in the differentiating and fully differentiated 3T3-L1 cells as well as human primary adipocytes by strongly inhibiting the expression of critical lipogenic enzymes, especially of FAS, which was achieved by directly suppressing the FAS promoter activities. Finally, nuciferine promoted the expression of FGF21 and ZAG in mature adipocytes, thus further contribute to inhibiting fat synthesis in the way of autocrine.

## Data Availability

The original contributions presented in the study are included in the article/Supplementary Material, further inquiries can be directed to the corresponding author.
