# Supplementary material for: Nuciferine Inhibited the Differentiation and Lipid Accumulation of 3T3-L1 Preadipocytes by Regulating the Expression of Lipogenic Genes and Adipokines
Source: Front Pharmacol. 2021 Mar 22;12:632236. doi: 10.3389/fphar.2021.632236 (PMC8025837; doi:10.3389/fphar.2021.632236)
Supplement: Supplementary file 1 [file Image1.jpeg]

Frontiers | Nuciferine inhibited the differentiation and lipid metabolism of 3T3-L1 preadipocytes by regulating the expression of lipogenic genes and adipokines | Pharmacology


- About
- Journals
- Research Topics
- Articles
- More

Submit

My Frontiers

Office

- TSOF
  - TSOF
  - Article Production

Typesetter 3

frontiersproduction@tnq.co.in

- Profile
- Settings & Privacy
- Help Center
- Logout

Submit

**Impact Factor 4.225** | **CiteScore 5.0**More on impact ›

|  |  |
| --- | --- |
| Frontiers in Pharmacology | Experimental Pharmacology and Drug Discovery |

Toggle navigation


Section


- (current)Section
- About
- Articles
- Research topics
- For authors 
  - Why submit?
  - Fees
  - Article types
  - Author guidelines
  - Review guidelines
  - Submission checklist
  - Contact editorial office
  - Submit your manuscript
- Editorial board

- *Article alerts*

Articles


**Suggest a Research Topic >**

- 44
  total views

 View Article Impact

**Suggest a Research Topic >**

##### SHARE ON

- Facebook

  0
- Twitter

  0
- LinkedIn

  0
- AddThis

  New


## Original Research ARTICLE

Front. Pharmacol.
| doi: 10.3389/fphar.2021.632236

# Nuciferine inhibited the differentiation and lipid metabolism of 3T3-L1 preadipocytes by regulating the expression of lipogenic genes and adipokines Provisionally accepted The final, formatted version of the article will be published soon. **Notify me**

Hanyuan Xu1,  Linjie Wang1, Kemin Yan1, 
Huijuan Zhu1, 
 Hui Pan1, Hongbo Yang1, 
 Meijuan Liu1 and  Fengying Gong1\*

- 1Peking Union Medical College Hospital (CAMS), China

Purposes: Nuciferine, a main aporphine alkaloid component found in lotus leaf (Nelumbo nucifera), has been demonstrated to possess the property of reducing fat mass and alleviating dyslipidemia in vivo. The purpose of this study is to explore the effects of nuciferine on the proliferation and differentiation of 3T3-L1 cells and further investigate the possible mechanisms.  
Methods: 3T3-L1 preadipocytes were treated with 0~20 μM nuciferine for 24~120 h, the cell viability was assessed using CCK8. 3T3-L1 preadipocytes and human primary preadipocytes were then induced differentiation and the effects of nuciferine on lipid metabolism in differentiating and fully differentiated adipocytes were observed by the methods of intracellular triglyceride (TG) assay, Oil Red O staining, RT-qPCR and western blot. Transient transfection and dual luciferase reporter gene methods were used to assess the effects of nuciferine on FAS promoter activities.  
Results: Nuciferine inhibited the proliferation of 3T3-L1 preadipocytes in a dose- and time-dependent manner. 20 μM nuciferine significantly attenuated lipid accumulation and reduced intracellular TG contents by 47.2%, 59.9% and 55.4% on the 3rd, 6th and 9th day of preadipocytes differentiation, respectively (all P<0.05). Moreover, the mRNA levels of PPARγ, C/EBPα, C/EBPβ, FAS, ACC, HSL and ATGL were notably decreased by 39.2~92.5% in differentiating preadipocytes (all P<0.05). In fully differentiated adipocytes, the mRNA levels of FAS, ACC and SREBP1 were remarkably downregulated by 22.6~45.2% compared with the controls (0 μM) (all P<0.05), whereas the expression of adipokines FGF21 and ZAG were notably promoted by nuciferine. Similar results were obtained in fully differentiated human primary adipocytes treated with nuciferine (all P<0.05). Further mechanism studies showed that 2.5~20 μM nuciferine significantly decreased FAS promoter activities in 3T3-L1 preadipocytes.   
Conclusions: Nuciferine inhibited the proliferation and differentiation of 3T3-L1 preadipocytes. The inhibitory effects of nuciferine on adipogenesis might be due to the downregulation of PPARγ, C/EBPα and C/EBPβ, which led to the reduction of intracellular lipid accumulation in 3T3-L1 cells and by downregulating the expression of critical lipogenic enzymes, especially of FAS, which was achieved by inhibiting the FAS promoter activities. Besides, nuciferine promoted the expression of adipokines in fully differentiated adipocytes.

Keywords: 
Nuciferine, differentiation, proliferation, Adipokines, Fatty acid synthase (FAS), 3T3-L1 preadipocytes

Received: 24 Nov 2020;
Accepted: 08 Feb 2021.

Copyright: © 2021 Xu, Wang, Yan, Zhu, Pan, Yang, Liu and Gong. This is an open-access article distributed under the terms of the Creative Commons Attribution License (CC BY). The use, distribution or reproduction in other forums is permitted, provided the original author(s) and the copyright owner(s) are credited and that the original publication in this journal is cited, in accordance with accepted academic practice. No use, distribution or reproduction is permitted which does not comply with these terms.

\* Correspondence: 
PhD. Fengying Gong, Peking Union Medical College Hospital (CAMS), Beijing, China, fygong@sina.com

Write a comment...

Add

##### COMMENTARY

##### ORIGINAL ARTICLE

##### People also looked at

**Suggest a Research Topic >**

×

#### Supplementary Material

  

There is no supplementary material currently available for this article

Loading supplemental data...

  

|  | File Name |  |
| --- | --- | --- |
|  | Table 1.docx |  |
|  | Data Sheet 1.docx |  |
|  | Image 1.JPEG |  |

  

Close

- About Frontiers
- Institutional Membership
- Books
- News
- Frontiers' social media
- Contact
- Careers
- Submit
- Newsletter
- Help Center
- Terms & Conditions
- Privacy Policy

© 2007 - 2021 Frontiers Media S.A. All Rights Reserved

### Privacy Preference Center

Our website uses cookies that are necessary for its operation. Additional cookies are only used with your consent. These cookies are used to store and access information such as the characteristics of your device as well as certain personal data (IP address, navigation usage, geolocation data) and we process them to analyse the traffic on our website in order to provide you a better user experience, evaluate the efficiency of our communications and to personalise content to your interests. Some cookies are placed by third-party companies with which we work to deliver relevant ads on social media and the internet. Click on the different categories' headings to change your cookie preferences. Click on "More Information" if you wish to learn more about how data is collected and shared.
More information

### Manage Consent Preferences

#### Strictly Necessary Cookies

Always Active

These cookies are necessary for the website to function and cannot be switched off in our systems. They are usually only set in response to actions made by you which amount to a request for services, such as setting your privacy preferences, logging in or filling in forms. You can set your browser to block or alert you about these cookies, but some parts of the site will not then work. These cookies do not store any personally identifiable information.

#### Analytics Cookies

Analytics Cookies

These cookies allow us to count visits and traffic sources so we can measure and improve the performance of our site. They help us analyse which pages are the most and least popular and see how visitors move around the site.    All information these cookies collect is aggregated and therefore anonymous.

#### Functional Cookies

Functional Cookies

These cookies enable the website to provide enhanced functionality and personalisation. They may be set by us or by third party providers whose services we have added to our pages. If you do not allow these cookies then some or all of these services may not function properly.

#### Advertising Cookies

Advertising Cookies

These cookies may be set through our site by our advertising partners. They may be used by those companies to build a profile of your interests and show you relevant adverts on other sites.    They do not store directly personal information, but are based on uniquely identifying your browser and internet device. If you do not allow these cookies, you will experience less targeted advertising.

### Back Button Performance Cookies

Vendor Search  Search Icon

Filter Icon

Clear

checkbox label label

Apply Cancel

Consent Leg.Interest

checkbox label label

checkbox label label

checkbox label label

Confirm My Choices
