# Supplementary material for: Nuciferine Inhibited the Differentiation and Lipid Accumulation of 3T3-L1 Preadipocytes by Regulating the Expression of Lipogenic Genes and Adipokines
Source: Front Pharmacol. 2021 Mar 22;12:632236. doi: 10.3389/fphar.2021.632236 (PMC8025837; doi:10.3389/fphar.2021.632236)
Supplement: Supplementary file 3 [file table1.docx]

# Supplementary Material

# Table S1: Primers for RT-qPCR

| Gene | Forward primer | Reverse primer |
| --- | --- | --- |
| m-FAS | GGAGGTGGTGATAGCCGGTAT | TGGGTAATCCATAGAGCCCAG |
| m-FGF21 | CTGCTGGGGGTCTACCAAG | CTGCGCCTACCACTGTTCC |
| m-ACC | GATGAACCATCTCCGTTGGC | GACCCAATTATGAATCGGGAGTG |
| m-SREBP1 | TGACCCGGCTATTCCGTGA | CTGGGCTGAGCAATACAGTTC |
| m-PPIA | GAGCTGTTTGCAGACAAAGTTC | CCCTGGCACATGAATCCTGG |
| m-ZAG | AGCAAAGGTTTTCCGAGGTTT | GAGACCCTGTAGTGTCCTTGTAA |
| m-HSL | CCAGCCTGAGGGCTTACTG | CTCCATTGACTGTGACATCTCG |
| m-ATGL | TTCACCATCCGCTTGTTG | AGTTCCACCTGCTCAGAC |
| m-C/EBPα | CAAGAACAGCAACGAGTACCG | GTCACTGGTCAACTCCAGCAC |
| m-C/EBPβ | CTTCAGCCCGTACCTGGAG | GGAGAGGAAGTCGTGGTGC |
| m-PPARγ | GACATTCCATTCACAAGAGC | TTCAGAATAATAAGGTGGAGATGC |
| h-PPIA | CCCACCGTGTTCTTCGACATT | GGACCCGTATGCTTTAGGATGA |
| h-FAS | AAGGACCTGTCTAGGTTTGATGC | TGGCTTCATAGGTGACTTCCA |
| h-FGF21 | ATGGATCGCTCCACTTTGACC | GGGCTTCGGACTGGTAAACAT |
| h-ACC | ATGTCTGGCTTGCACCTAGTA | CCCCAAAGCGAGTAACAAATTCT |
| h-SREBP1 | ACAGTGACTTCCCTGGCCTAT | GCATGGACGGGTACATCTTCAA |
| h-ZAG | GCTTACCTGGAGGAGGAGTG | TTCCCTGGGTAGAAGTCGTAG |
| h-HSL | TCAGTGTCTAGGTCAGACTGG | AGGCTTCTGTTGGGTATTGGA |

m: mouse

h: human
